# Patient No-Show Prediction: A Systematic Literature Review

**DOI:** 10.3390/e22060675

**Published:** 2020-06-17

**Authors:** Danae Carreras-García, David Delgado-Gómez, Fernando Llorente-Fernández, Ana Arribas-Gil

**Affiliations:** 1 Department of Statistics, University Carlos III of Madrid, 28911 Leganés, Spain; dcarrera@est-econ.uc3m.es (D.C.-G.); felloren@est-econ.uc3m.es (F.L.-F.); 2 UC3M-Santander Big Data Institute, University Carlos III of Madrid, 28903 Getafe, Spain; aarribas@est-econ.uc3m.es

**Keywords:** patient no-show, prediction, systematic review

## Abstract

Nowadays, across the most important problems faced by health centers are those caused by the existence of patients who do not attend their appointments. Among others, these patients cause loss of revenue to the health centers and increase the patients’ waiting list. In order to tackle these problems, several scheduling systems have been developed. Many of them require predicting whether a patient will show up for an appointment. However, obtaining these estimates accurately is currently a challenging problem. In this work, a systematic review of the literature on predicting patient no-shows is conducted aiming at establishing the current state-of-the-art. Based on a systematic review following the PRISMA methodology, 50 articles were found and analyzed. Of these articles, 82% were published in the last 10 years and the most used technique was logistic regression. In addition, there is significant growth in the size of the databases used to build the classifiers. An important finding is that only two studies achieved an accuracy higher than the show rate. Moreover, a single study attained an area under the curve greater than the 0.9 value. These facts indicate the difficulty of this problem and the need for further research.

## 1. Introduction

The existence of patients who do not keep their appointments, commonly referred to as no-shows, is currently one of the main problems of health centers. The absence of patients from their appointments causes the under-utilization of the center’s resources, which extends the waiting time of other patients. No-shows also have an economic impact on health facilities limiting future staff recruitment and the improvement of the center’s infrastructure. As an example, considering only the primary care centers in the United Kingdom, the number of missed appointments exceeds 12 million [1]. Moore et al. [2] reported that the percentage of no-shows and cancellations represented 32.2% of the scheduled time in a family planning residence clinic. In terms of economic losses, they reported that the decrease in the health center’s annual income ranges from 3% to 14%. In the United Kingdom, the annual economic cost caused by non-shows is 600 million pounds [3]. Besides the problems caused to the health center and the rest of the patients, missing an appointment can cause serious health problems for the no-show patients [4].

In order to reduce these negative effects, health centers have implemented various strategies including sanctions and reminders. However, besides the fact that various articles question their efficiency [5,6], these strategies have a significant cost associated with them. On the one hand, sanctions may limit the access to patients with restricted incomes to medical centers [7]. On the other hand, reminders have an economic impact that has been estimated at 0.41 euros per reminder [8].

During the last decades, a significant number of scheduling systems have been developed to provide an alternative to these strategies. These systems aim to achieve better appointment allocation based on the patient no-show prediction. They are described in several review articles, such as Cayirli and Veral [9] and Gupta and Denton [10], and more recently by Ahmadi-Javid et al. [11]. An important aspect to point out is that the efficiency of these systems depends mainly on two elements: the discriminatory capacity of the predictors and the classification technique used to estimate the probabilities.

Regarding the first of these two elements, several research works have been carried out to discover which are the best predictors that discriminate the patients who attend their appointments from those who do not. These investigations have led to the identification of a significant number of predictors, such as the percentage of previous no-shows, lead time, diagnosis, or age. A recent review of the literature by Dantas et al. [12] has identified more than 40 potential predictors.

In contrast to the research conducted in the identification of good predictors and in the construction of accurate scheduling systems that have been conducted since the 1960s, research on the development of predictive models has mainly been carried out in the last decade. This is primarily since, until the recent availability of Electronic Health Records (EHR), there were no databases of sufficient size to build these models accurately. It is important to note that building accurate classifiers is essential for the scheduling system to work effectively. However, obtaining these predictions is still an unsolved problem on which a significant number of publications are appearing.

In this work, a systematic review is carried out to establish the state-of-the-art in no-show prediction. The review aims to identify the models that have been proposed along with their strengths and weaknesses. To accomplish this, besides identifying each of the different techniques, various elements such as the characteristics of the database, the protocol employed to evaluate the model, or the performance obtained are analyzed. The review also identifies which are the most widely used predictors in the literature.

The rest of the article is structured as follows. In Section 2, the bibliographic search protocol is presented. This includes the bibliographic databases, the search criteria, the exclusion criteria, and the variables that will be extracted from each of the selected articles. Next, in Section 3, the selected articles are described, grouping them according to the proposed technique, and exposing their most relevant contributions. The article ends in Section 4 where a discussion on the findings is made and the conclusions are presented.

## 2. Methods

This section presents the search strategy, the screening criteria as well as the methodology followed to analyze the selected studies. This is done using the Preferred Reporting Items for Systematic Reviews and Meta-Analyses (PRISMA) guidelines [13].

### 2.1. Bibliographic Databases and Search Criteria

Two major bibliographic databases were used to search for the articles: Scopus (which covers the 100% of MedLine) and Web of Knowledge. The search terms that were used are shown in Table 1.

The purpose of this search is to find those articles that aim to identify patients who will not show up for their appointments. Therefore, initially, the search is restricted to those articles that focus on no-shows by including the keyword *no-show* or its synonyms. To discard those articles dealing exclusively with non-attendance factors or scheduling systems, the keyword predict was added to the search. In both cases, the term * is added to include variations of the keywords. These queries were applied to the title, the abstract, and keywords of the articles. The search, which covered the period from January 1980 to January 2020, was carried out on both databases independently and the results were merged as explained in the following section.

### 2.2. Study Selection

**Figure 1:**
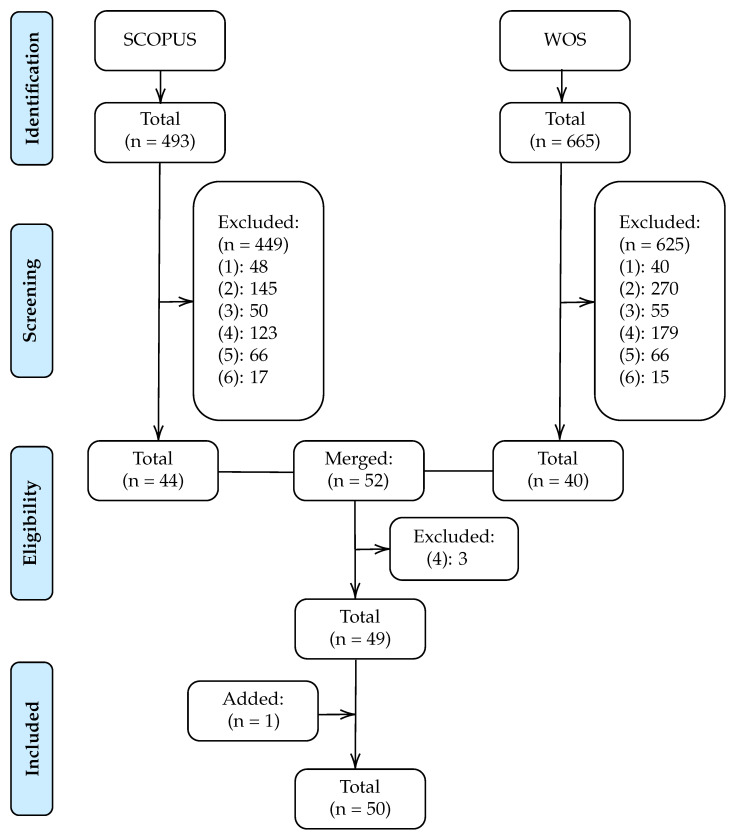
Flow diagram of study selection.

The articles analyzed in this systematic review were selected through a four-stage procedure, as shown in Figure 1.

In the first stage, potentially relevant articles were identified from applying the above query to the SCOPUS and WOS databases. The search on Scopus resulted in 493 potential articles while the WOS identified 665.

Next, in the screening stage, the selected articles that did not address the prediction of no-shows were discarded. For this purpose, based on abstracts reading, articles that met any of the following six criteria were excluded:

1. The article does not focus on the field of health. For example, articles that focus on predicting no-shows on airplanes or in restaurants.
2. The article does not focus on predicting no-shows. For instance, articles in the health field that focus on predicting a dependent variable other than no-shows such as treatment non-adherence.
3. The article focuses exclusively on developing a scheduling system.
4. The article focuses only on identifying which factors are related to the non-attendance of the patient without focusing on classification rates or other performance measures.
5. The article analyses the incidence of a factor in the no-show rate. For example, the impact of sending phone reminders on reducing the no-show rate.
6. The article provides only descriptive statistics on the relationship between the factors and the no-show dependent variable.

In the third stage, articles from both searches were merged, eliminating duplicates and discarding those that met some of the above exclusion criteria after reading the full article. After this stage, 49 articles were identified.

Finally, an article was added analyzing the references of the selected articles, obtaining a total of 50 articles.

### 2.3. Summary Measures

Each of the selected articles was analyzed in detail with the aim of extracting the value of the following variables:

(i) Year of publication.
(ii) *Characteristics of the database.* Number of patients, number of appointments and duration of the data collection.
(iii) *No-show rate*. Percentage of no-show on the whole database.
(iv) *Set-up.* Performance evaluation framework used. It is given by a triplet [x, y, z] that contains the data proportion employed for train, validation, and test, respectively, or by “CV” if a cross-validation framework was employed.
(v) *New patients*. Indicate if patient’s first visit is included in the analysis.
(vi) *Prediction model.* Classification model used in the classification problem.
(vii) *Performance Measures.* Metrics used to evaluate the model performance and its value.
(viii) *Feature selection.* Whether or not variable selection was applied, and if so, which technique was used. This is complemented with information on the most important variables found in each study.
(ix) *Service:* If the study was conducted in a primary care center, in a specialty, or both.

This information was collected in three concept matrices, as shown in Table 2, Table 3 and Table 4. In the first one, all the previous descriptions are presented. For studies reporting more than one performance measure, the value of the most relevant one is provided. The second one shows the most important predictors reported in each article. Finally, the third one reports the performance measures used in each article.

## 3. Results

**Figure 2:**
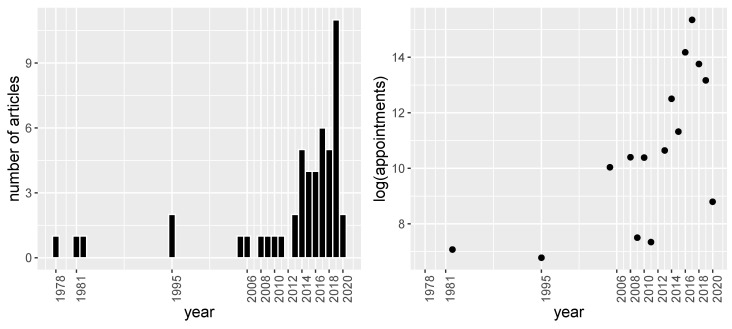
(**a**) Number of articles published per year. (**b**) Average number of appointments per year used in the databases on a logarithmic scale.

Before starting to describe each of the articles, we illustrate in Figure 2 the distribution of the publication by year and the size of the dataset. In Figure 2a it can be seen that most of the articles have been published in the last decade. This shows the current interest in the no-show prediction problem. Another interesting aspect, shown in the right panel of Figure 2b, is the size of the databases used. It can be seen that there has been a rapid growth in the last years (with a statistically significant exponential trend) that could be explained by the recent availability of electronic health records.

After analyzing the distribution of the number of articles published annually and the evolution of the size of the databases, the most relevant aspects of each selected articles are described below. The exposition is carried out by grouping the articles that use the same predictive model. Within each of these groups, the articles are presented chronologically. The different models considered are regression models (30 articles), tree based models (nine articles), neural networks (three articles), Markov based models (one article), Bayesian models (three articles) and ensemble/stacking models (four articles), with some of the articles implementing more than one predictive model.

### 3.1. Regression Models

Without a doubt, logistic regression (LR) is the most commonly used technique to predict missing attendance. Among the selected studies, Dervin et al. [14] was the first author to use multiple LR to identify no-shows in 1978. They used 10 predictors obtained from a small sample of 291 family practice center patients. Unfortunately, the results obtained were disappointing. Applying a combination of LR and linear discriminant (LD), they were only able to achieve an accuracy of 67.4% in a sample with an attendance rate of 73%.

Subsequently in 1982, Goldman et al. [15] conducted a similar study in which a multiple LR was used to predict no-shows in a sample of 376 patients from a primary care center. Among the 25 initial predictors, they identified as the most significant variables age, race, number of no-shows in the previous 12 months and whether the patient had psycho-social problems. One advantage of this study is that the authors split the observations into training and test sets in order to obtain more representative results. However, they did not provide any performance indicators in the test sample. They only stated that the obtained results were acceptable. A further limitation of this work is that they remove the first visits in the study, which reduces notably the complexity of the problem.

Another article in which a multiple LR model was used is Lee et al. [16] in 2005. In this article, a database of about three million appointments was used. However, only the most recent appointment of each patient was used to train the model, reducing the previous number to about 22,000 appointments. The authors reported an accuracy of 73%, which is slightly lower than the attendance rate of 79%. An important limitation of the study is that the data were not divided into training and test in the experiments. Also, they did not include the patient’s first visit to the analysis.

In 2006, Qu et al. [17] used LR considering interactions between the six factors used. The results reported were promising (Root mean squared error of 3.6%). However, as with other previous work, the fact that the set-up used did not split the data into training and test sets raises the question of how these results would look in a more realistic scenario.

In 2010, Daggy et al. [7] carried out one of the first works in which the estimation of the no-show probabilities is incorporated into a scheduling system. Regarding the performance of the model, using a training and test set-up, the authors reported an AUC of 0.82 in a database containing a 15.2% no-show rate. With these probabilities, the scheduling system achieved an expected benefit per patient of $100.

In 2011, Alaeddini et al. [18] proposed to combine global predictions, which use information about the whole population in terms of a set of variables, together with individual predictions, which only use the past no-show history of patients, to estimate the probabilities. In their model, LR obtained initial estimates of the probability of no-show for each patient. These were later improved through a Bayesian update (BU) and a weighting optimization. Unfortunately, although they reported an accuracy of 79.9% in a training and test set-up, it is not possible to assess the performance of this work since they did not provide the no-show rate. The authors subsequently extended this model to the multinomial case to include cancellations [19].

In 2013, Cronin et al. [20] used new and follow-up appointments to predict no-shows through LR in a dermatology department. To do so, they only included the variables that were significant in the univariate models. They concluded that patients who are younger, have been waiting a long time, or have less comprehensive insurance are more prone to miss an appointment. However, as Goldman et al. [15], they did not provide any performance measures.

In 2014, Norris et al. [21] investigated whether analyzing jointly no-shows and cancellations would improve no-show predictions. This question was addressed with both multinomial LR and decision trees. Their experiments showed that the best results were obtained using a binary LR that took into account only no-shows. This approach obtained an accuracy of 81.5% in identifying no-shows. However, this figure did not reach 91.1% that would be obtained if all patients were classified as show. Similarly, Ma et al. [22] applied an LR in which the accuracy did not exceed the one that would be obtained by a classifier that labels all the observations as attended (65% vs. 80.8%).

The same year, Huang and Hanauer [23] approached the problem from a planning systems perspective. They claimed that a false positive is a more serious problem than a false negative in no-show prediction. Classifying a patient as a no-show when he/she actually attends causes serious issues to the overbooking planning systems. For example, it increases the patient’s time in the clinic and adds to the cost of the doctor’s extra time. This fact was taken into account to select the threshold. Despite obtaining an accuracy of 86.1%, in a database with an attendance rate of 88.8%, the waiting time of patients for medical services was reduced by 6–8%.

In 2015, Woodward et al. [24] used LR to predict the no-shows in HIV-infected patients. The relevance of this work is that variables such as the presence of drugs or heterosexual contact were identified as significant while age, which is frequently used in other work, was considered not relevant. This result seems to indicate that variables that are highly informative in one setting may not be so in another. The limitation of this study is that they did not present any performance measures. This was not the case for Torres et al. [25], which reported an AUC of 0.71 in their study. Their LR model was conducted by including only those variables that were significant in the individual models.

In this year, the first articles considering feature extraction were published. Blumenthal et al. [26] incorporated for the first time variables obtained through natural language processing techniques from doctors’ notes. In particular, they generated a variable called non-adherence rate. These authors reported an AUC of 0.702 in a database with a no-show rate of 13.69%. A year later, Peng et al. [27] analyzed the possibility of obtaining discriminative variables by applying principal component analysis to a set of 19 predictors associated with weather. However, the two extracted variables were not significant.

In 2016, several research articles considered the time dependence. Until then, previous research works considered the appointments within the same patient as independent observations. Harris et al. [28] built a model called “sums of exponential for regression” (SUMER) that only considered the attendance record of each patient. Two limitations of this model are the need to have a large number of appointments for each patient, and that it discards the information of clinical and socio-demographic variables. In another article, Huang and Hanauer [29] sequentially built several LR in which each of them predicted the attendance at the next visit of patients with the same number of previous appointments. In addition to the explanatory variables, the model for patients with n appointments includes the patient’s attendance record and the probabilities estimated by the previous *i* models (i = 1, …, n − 1). They reported that the AUC increased slightly as more information became available, obtaining a maximum AUC of 0.706 in the 19th model. An article published that year by Kurasawa et al. [30] deserves special attention. In this work, an LR with feature selection is conducted by means of an L2 regularization. This work is highlighted because of the high value of the AUC obtained (0.958). The authors claim that this outstanding result is due to the inclusion of an important number of variables related to the patient’s diagnosis.

In 2017, Alaeddini and Hong [31] adopted the previous idea in Kurasawa et al. [30] of performing feature selection using penalizers. They proposed a multi-way multi-task learning model based on multinomial LR and an L1/L2 regularization. Using this model, the authors achieved an accuracy close to 80%. However, the limitation of this work is the size of the database, which contained only 410 appointments. As an alternative, Goffman et al. [32] considered a traditional stepwise feature selection approach instead of penalizers. An important contribution of this work is that the variable describing the patient’s attendance record was obtained through an empirical Markov model based on the ordered series of no-shows of the last 10 appointments. Stepwise LR was also used by Harvey et al. [33] and more recently by Gromisch et al. [34]. An interesting article is Mieloszyk et al. [35], which was the first work that used a five fold cross-validation to perform the feature selection in LR.

In 2018, only one article addressed the prediction of no-shows from a regression-based perspective. Specifically, Ding et al. [36] asked whether it is preferable to create several local models that consider the different combinations of clinics and specialties or a single global model. For this purpose, they built 420 multivariate LR models with L1 regularization for feature selection. They observed that the local models fitted the data better although they exhibited a lot of variability. For this reason, they concluded that there are no simple rules for determining whether local or global models are preferable, but this decision depends on the data being analyzed.

In 2020, Lin et al. [37] followed the research line proposed by Ding et al. [36] consisting of the development of several local models. In this work, 475 LR models were built, one for each physician. In order to conduct the feature selection and estimate the model’s parameters, the authors considered Lasso-based Bayesian modeling and automatic relevant determination. The best approach was achieved using Lasso-based Bayesian, which attained AUC values ranging between 0.70 and 0.92. An approach that lies between building a single global model and many local models is to use a mixed effects logistic regression model (MELR). This approach was proposed by Lenzi et al. [38] and Li et al. [39]. In particular, the former, which determines the most parsimonious model based on the Akaike information criterion, groups by patient and provider, while the latter groups by patient and appointment confirmation. In these studies, AUCs of 0.81 and 0.886 were reported, respectively. Another approach that has been considered to predict no-show is probit regression in Ahmad et al. [40], obtaining an AUC of 0.7. Other research works used LR in 2019, like those of Chua and Chow [41] and Dantas et al. [42]. However, these studies focus more on feature selection based on a filter method. In both cases, modest results were obtained. Specifically, Dantas et al. [42], which did not achieve an accuracy higher than the classification rate.

### 3.2. Tree Based Models

Decision Trees (DTs) represent the most widely used method after regression models. The first work using this technique to identify no-shows was Dove and Schneider [43] in 1981. They analyzed whether the use of individual characteristics of the patient was better than considering the average rate of the clinic to predict missing appointments. For this purpose, data from 1333 patients from 35 clinics were used for building a DT using the automatic interaction detection feature algorithm. The authors, based on the mean absolute error of 14.8%, claimed that the use of patient characteristics was as accurate as the average clinical rates.

The next study that used DTs to predict no-shows was Bean and Talaga [44] in 1995. In this article, the tree was constructed using a chi-squared automatic interaction detector (CHAID). A drawback of this paper is that, although the authors show the probability of no-show for each patient from the created rules, they do not provide any measure of accuracy.

In 2009, the first scheduling system that considered individualized predictions of patient attendance was developed by Glowacka et al. [45]. These estimates were determined using an association rule mining (ARM) that can be viewed as a DT. A limitation of this method is that it does not classify all observations. Specifically, in this study, the 13 rules generated classified only 390 of the 1809 observations available. Another drawback is, as in the previous work, the lack of a performance measure of classification.

Later in 2014, Lotfi and Torres [46] used several algorithms to build DTs which predict no-shows in the most recent appointment. The algorithms considered were CHAID, Exhaustive CHAID, Classification and Regression Trees and Quick, Unbiased, Efficient Statistical Tree. A drawback is that the best model had an accuracy of 78%, which is below the attendance rate of 84%. As shown by Glowacka et al. [45], the estimated probabilities were incorporated into a scheduling system, increasing center utilization from 46% to 72.9%.

In 2017, Devasahay et al. [47] compared the performance of DTs and LR in predicting no-shows. They reported a good specificity at the cost of a very poor sensitivity (4% vs. 99%) for specific thresholds or a poor prediction in these two performance measures (23% vs. 24%) for both DTs and LR. The authors justified these results due to class imbalance.

Between 2018 and 2019, several works analyzed the advantages of using DTs over LR. As Devasahay et al. [47], Alloghani et al. [48] obtained good specificity at the cost of very poor sensitivity (3% vs. 99%) or poor prediction in the above two performance measures (25% vs. 22%) for specific thresholds values on both models. Better results were obtained by Praveena et al. [49] in which the accuracy of DTs was higher than the one attained by the LR and also exceeded the attendance rate. In 2019, AlMuhaideb et al. [50] tried to solve the problem from a different point of view. They used two algorithms based on information gain to build the tree (JRip and Hoeffding). However, none of the two building techniques achieved an accuracy that exceeded the attendance rate.

A very recent application of DTs to predict no-show is Aladeemy et al. [51] in 2020. In this study, the authors considered different techniques such as DTs, random forest, k-nearest neighbors, support vector machines, boosting, naïve Bayes, and deep learning. They showed that the technique that achieved the best results was DTs. The novelty of this work is that the variables were selected through a metaheuristic called Opposition-Based Self-Adaptive Cohort Intelligence (SACI). This work, together with those published in the previous year, indicates the attention that DTs are receiving in the prediction of no-shows in recent years.

### 3.3. Neural Networks

Neural networks (NN) is currently one of the techniques for binary classification that receives the most attention in the field of artificial intelligence. The first article that used this methodology to predict no-shows dates back to 1995 by Snowden et al. [52]. In this article, the authors used a backpropagation NN with a hidden layer of five neurons to obtain a correct classification of 91.11% in a database with approximately 20% of no-shows. However, a drawback of this work is that only the 190 patients with complete data from a database of 300 patients were used. Another limitation is that the network did not make any predictions in 5% of the data. In addition, because they conducted a single validation experiment, the results may not be reliable.

In 2014, Dravenstott et al. [53] used NN to predict no-shows in a database of three million observations obtained from primary care and endocrinology departments. A network with two hidden layers was trained for each department, which obtained an accuracy of 83% and 87%, respectively. However, none of these networks surpassed the attendance rate.

Recently, in 2019, Dashtban and Li [54] developed a sparse stacked denoising autoencoder for predicting patient non-attendance that handled incomplete data. The sparsity of the model was achieved by means of a constraint based on Kullback–Leibler divergence (relative entropy). For this purpose, one part of the network consisted of an autoencoder. The network was trained with a database of 1.6 million appointments, obtaining an AUC of 0.71. They also reported an accuracy of 69%, but it is not possible to evaluate the performance of this network since the no-show rate was not reported.

### 3.4. Markov Based Models

In 2008, Chariatte et al. [5] proposed a double chain Markov model (MM) as an alternative to the widely used LR to account for the temporal dependence. The authors expected better performance than with LR and a better characterization of no-shows. However, they did not report any performance measures. A drawback of this research work is that they only included patients with more than three appointments in the study, which significantly reduces the complexity of the problem.

### 3.5. Bayesian Models

The first study that used a Bayesian network (BN) to predict non-attendance was conducted in 2013 by Levy [55]. The authors considered different thresholds that resulted in a sensitivity ranging from 60% to 65% and a specificity ranging from 37% to 42%, in a database with a no-show rate of 16%.

Later, in 2018, Mohammadi et al. [56] compared the performance of a Naïve Bayes classifier with respect to LR and NNs. In order to ensure independence between observations, a database of 73,811 observations corresponding to the last visit of each patient was used. The model that obtained the best performance was the Naive Bayes Classifier with an AUC of 0.86. Also that year, Topuz et al. [57] proposed the Tree Augmented Bayesian Network, which is an improved version of Naïve Bayes. This BN uses the variables that are previously selected by the Elastic Net algorithm. The authors showed that this network improved the results obtained by an LR.

### 3.6. Ensemble/Stacking Methods

Ensemble and stacking methods are techniques that combine predictions from several classifiers. The difference between these two techniques is that the former makes a weighted sum of these predictions, while the latter unifies the predictions by means of a classifier.

In 2017, Lee et al. [58] used gradient boosting (GB) for the first time to combine the predictions from various decision trees. Using 60 variables obtained through text mining together with various socio-demographic variables, they reported an AUC of 0.832.

The following year, Elvira et al. [59] used GB to minimize the problem of class imbalance. However, their results were modest since the AUC was lower than 0.75. The authors concluded that they did not have enough information to predict missing appointments accurately. In turn, Srinivas and Ravindran [60] developed a stacking model to predict the no-show in a primary care center. This model, which used an LR to combine the predictions obtained from NNs, random forest (RF) and stochastic GB attained an AUC of 0.846.

In 2019, Ahmadi et al. [61] used a stacking approach for which the diversity of the model was achieved providing different variables to an RF model. These variables were selected through a genetic algorithm.

## 4. Discussion and Conclusions

In this work, a systematic review has been carried out on the prediction of patient no-show. The relevance of the problem can be observed in the fact that 41 of the articles on no-show prediction (82% of the total) have been published in the last 10 years (and 32, that is 64% of the total, in the last five years). The review has identified several factors that influence the results reported in each of the studies analyzed. These factors include the choice of the predictive model, the features used by these models, the variable selection, the performance assessment framework, the class imbalance together with the performance measure, the intra-patient temporal dependence, and whether the experiments take into account the first visits or not. The main findings for each of these factors are described in detail below.

**Predictive models.** The revision found out that the most widely used algorithm was LR, which appears in 30 articles, that is, more than 50% of the total. This can be explained by the fact that the early works were focused on identifying the most influential factors in patient no-shows, in which the LR plays a primordial role. The second most frequent predictive model is DTs, used as a primary technique in 10 articles (20% of the total). Among the different models, the LR with L2 regularization proposed by Kurasawa et al. [30] stands out, achieving an AUC of 0.958. Another work that deserves special attention is Snowden et al. [52], which used NN, reaching an accuracy of 91.11% in a database with an attendance rate of 80%. With the current explosion of deep networks and the growth in databases, this methodology is a promising line of research.

**Features.** As both Deyo and Inui [62] and Dantas et al. [12] indicated, there are no universal variables in the no-show patient databases. The most appropriate variables depend, for example, on the population under study or the specialty. However, as shown in Table 3, some variables show a discriminatory capacity in the majority of the studies. Variables such as age, gender, insurance, distance, weekday, visit time, lead time, and no-show appeared in at least half of the studies. Among these variables, previous no-shows (along with the number of previous appointments) have been reported as the most significant. This shows the importance of including the patient’s history in the study and reaffirms the intra-patient dependence on observations. Although on a smaller scale, variables such as race, marital status and visit type (first/follow-up) have also been frequently used. A limitation of existing studies is that, in many cases, using a variable depends on its availability in the EHR.

**Feature selection.** Another aspect worth mentioning is whether the studies perform feature selection, as this can significantly influence the performance of predictive models. As a general rule, the addition of variables with low predictive capacity reduces the generalization of the results. According to Guyon and Elisseeff [63], feature selection techniques can be divided into three main groups: filter, wrapper, and embedded. The filtering methods, which are the most used in the analyzed articles, select the variables before passing them to the predictive model. The majority of the works that employed a filtering technique used univariate models to select significant variables [15,20,21,23,24,25,26,29,41,42,53]. On the other hand, wrapper methods evaluate multiple models that are created with different combinations of variables. The most used technique within the wrapper methods was stepwise feature selection [7,32,33,34,38]. Other techniques are metaheuristics such as genetic algorithms [61] or Opposition-Based Self-Adaptive Cohort Intelligence [51]. Finally, embedded methods incorporate the selection of variables within the model itself. In this category, the most used techniques were decision trees [43,46,47,50,59] and penalized regression [30,31,36,37,57]. In fact, studies that applied penalized LR such as Kurasawa et al. [30] and Lin et al. [37] present the best adjustment measures.

**Performance evaluation framework.** A very important aspect is the experimental design since it conditions the generalization of the results. In 13 studies (26% of the total), the performance of the model was evaluated on the same data that were used to train it. It is a well known fact that this approach is prone to overfitting the data, which results in a drastic decrease in accuracy when the developed classifier is used in future data. Thirty-one of the articles (62% of the total) conducted a single validation in which the data were divided into training and testing. The disadvantage of this approach is that there is no guarantee that the easiest-to-classify observations might be in the test set, which leads to overconfident results. Only six studies (12% of the total) performed a repeated validation or a k-fold cross-validation. These number indicates that the results reported may be not realistic with new datasets.

**Performance measures.** An important aspect to point out from our analysis is that the no-show prediction performance is evaluated very differently across studies. In particular, 44 out of the 50 articles report at least one measure of performance. Among these, the most commonly used metric was AUC, included in 29 of the works. Of these 29 works, a single study obtained an AUC value larger than 0.9 and only six articles (near to 20%) reported an AUC higher than 0.85. The second most used performance measure was accuracy, reported in 21 articles. Following, 17 studies reported specificity and sensitivity, six reported PPV and NPV, and four used recall and precision as performance metrics. Finally, four studies reported an error measure (MAE, MSE or RMSE), and only one proposed to use F-measure and another one to use G-measure (see the next paragraph). This heterogeneity on the use of performance measures makes difficult the comparison of results across studies. The detailed information of the performance measures used can be found in Table 4.

**Imbalance problem.** As already mentioned, the class imbalance is a common characteristic of all the studies analyzed. A fact observed in several studies is that the accuracy obtained was lower than the attendance rate [14,16,21,22,23,42,46,50,53,59]. In particular, accuracy exceeds the attendance rate in only 5 of the 15 studies reporting these two values. This low performance could be partly due to the class imbalance that biases the different algorithms to predict each observation as a show. In the analyzed studies, 26 of the works report a no-show rate lower than 20%, which represents 68% of the 38 articles that presented this index. Several approaches have been proposed in the literature to deal with class imbalance in binary classification (see [64] for an overview). They can be categorized in three groups: (1) those based in training set data transformations aimed at reducing the imbalance between the classes (by undersampling or oversampling the majority or minority class, respectively), (2) those based in the use of specific algorithms that take into account the prior imbalanced class distribution and (3) hybrid approaches combining (1) and (2). Among the analyzed articles, only the cost-sensitive method proposed by [29] and the ensembles/stacking methods can tackle class imbalance. They fit into the second of the three above mentioned groups, that is, the algorithm-level approaches. Among these works, only Elvira et al. [59] relates the classifier choice with the imbalance problem. The authors of this study also pointed out that accuracy was not an adequate performance measure and proposed to use the AUC. Alternatively, Kurasawa et al. [30] proposed to use the F-score and Topuz et al. [57] proposed the G-measure.

**Temporal dependence.** An important aspect is the intra-patient temporal dependence of the observations. Several authors avoid this problem using only the last appointment (most recent) to train the model [7,16,21,23,25,32,46,56]. However, this approach results in a loss of information. Only 7 of the 50 analyzed articles include the intra-patient temporal dependence in the model. This dependence was incorporated using different approaches including Markov chains [5], weighting observations by their temporal closeness [18,19], using an exponential sum for regression [28], building various LR based on the number of previous visits [29], or using a MELR [38,39]. The last approach provides a promising approximation in the resolution of the problem since it allows to unify the behavior of the patient, the socio-demographic variables, and the environmental variables.

**New patient.** One element that significantly affects the results is the inclusion of new patients in the analysis. At the time of the first visit of the patient, the available information is very limited. Only some environmental variables are available (e.g., month, day and time of the appointment) and perhaps some socio-demographic variables (age and sex of the patient). This limitation makes it very difficult to predict missing attendance on the first visit. Different authors have addressed this problem by means of different techniques such as not including in the analysis patients who do not have a certain number of previous visits [5,28], not including the first appointment in the study [7,15,16,21,30,44,53], or including a variable that indicates whether the appointment corresponds to the first visit [14,17,20,23,27,29,32,34,40,41,42,43,48,50,51,52,56,58,59,60].

To conclude, the above discussion has shown that the identification of patients who do not attend their appointments is a challenging and unsolved problem. As it was shown above, this can be observed in the fact that only five articles attained an accuracy higher than the no-show rate. This is a consequence of several pitfalls. Firstly, the researchers only had access to a limited number of predictors with low discrimination capacity and, in addition, those were not the same for all the researchers. Moreover, many studies were conducted with databases consisting of a small number of patients, which limited the information provided to the classifiers. However, the recent availability of more informative databases obtained from EHR opens up new research opportunities. These current databases containing records of hundreds of thousands of appointments allow the use of modern predictive techniques such as deep neural networks or novel binary classification algorithms for high-dimensional settings, such as [65,66]. A second research line consists of developing and incorporating strategies that reduce the negative effects of class imbalance. For instance, the use of sampling techniques, cost-sensitive approaches, or the previously commented ensemble models might improve the performance of the selected classifier. A third possibility is the incorporation of intra-patient temporal dependence, which would allow a better characterization of the patients’ behavior by unifying their previous attendance records, their socio-demographic characteristics, and the environmental variables. These strategies could lead to obtaining more accurate predictions that, when incorporated into scheduling systems, will reduce the economic losses suffered by health centers and the waiting time for access to the medical services.

## Figures and Tables

**Table 1:** Consultation employed.

	Keywords
	non-attendance OR missed-appointment* OR no-show*
(i)	OR broken-appointment* OR missed-clinic-appointment*
	OR appointment-no-show*
(ii)	predict*
(iii)	(i) AND (ii)

**Table 2:** Summary of studied articles.

Articles	Patients	Appointments	Months	Service	No-Show Rate	Set-Up	New Patient	Feature Selection	Model	Performance Measures
Dervin et al., 1978	291	-	×	Primary Care	27	[1,0,0]	×	-	LR, LD	67.4 (ACC)
Dove and Schneider, 1981	1333	-	12	Specialty	24.5	[1,0,0]	×	embedded	DT	14.8 (MAE)
Goldman et al., 1982	376	1181	6	Primary Care	18	[2/3,0,1/3]	-	filter	LR	-
Snowden et al., 1995	190	-	6	Specialty	20	[3/4,0,1/4]	×	-	NN	91.11 (ACC)
Bean and Talaga, 1995	-	879	4	Both	38.1	[1,0,0]	-	-	DT	-
Lee et al., 2005	22,864	22,864	48	Specialty	21	[1,0,0]	-	-	LR	0.84 (AUC)
Qu et al., 2006	-	-	24	Primary Care	-	[1,0,0]	×	-	LR	3.6 (RMSE)
Chariatte et al., 2008	2193	32,816	96	Specialty	-	[1,0,0]	-	-	MM	-
Glowacka et al., 2009	-	1809	9	Both	-	[1,0,0]	×	-	ARM	-
Daggy et al., 2010	5446	32,394	36	Specialty	15.2	[2/3,0,1/3]	-	wrapper	LR	0.82 (AUC)
Alaeddini et al., 2011	99	1543	2	-	-	[1/3,1/3,1/3]	×	-	LR-BU	79.9 (ACC)
Cronin et al., 2013	-	41,893	22	Specialty	18.6	[1,0,0]	×	filter	LR	-
Levy, 2013	4774	-	12	Specialty	16	[1/2,1/4,1/4]	×	-	BN	60–65 (Sens)
Norris et al., 2014	88,345	858,579	60	Primary Care	9.9	[3/5,0,2/5]	-	filter	LR, DT	81.5 (ACC)
Dravenstott et al., 2014	-	103,152	24	Both	9.1	[0.60,0.15,0.25]	-	filter	NN	87 (ACC)
Lotfi and Torres, 2014	367	367	5	Specialty	16	[0.55, 0, 0.45]	×	embedded	DT	78 (ACC)
Huang and Hanauer, 2014	7988	104,799	120	Specialty	11.2	[4/5,0,1/5]	×	filter	LR	86.1 (ACC)
Ma et al., 2014	-	279,628	6	Primary Care	19.2	[1/3,1/3,1/3]	×	-	LR, DT	65 (ACC)
Alaeddini et al., 2015	99	1543	2	-	22.6	[1/3,1/3,1/3]	×	-	LR-BU	0.072 (MSE)
Blumenthal et al., 2015	1432	-	22	Specialty	13.69	[0.78,0,0.22]	×	filter	LR	0.702 (AUC)
Torres et al., 2015	11,546	163,554	29	Specialty	45	[7/10,0,3/10]	×	filter	LR	0.71 (AUC)
Woodward et al., 2015	510	-	8	Specialty	27.25	[1,0,0]	×	filter	LR	-
Peng et al., 2016	-	881,933	24	-	-	[1,0,0]	×	-	LR	0.706 (AUC)
Kurasawa et al., 2016	879	16,026	39	Specialty	5.8	10 Fold CV	-	embedded	L2-LR	0.958 (AUC)
Harris et al., 2016	+79,346	4,760,733	60	-	8.9	[1/10,0, 9/10]	-	-	SUMER	0.706 (AUC)
Huang and Hanauer, 2016	7291	93,206	120	Specialty	17	[2/3,0,1/3]	×	filter	LR	0.706 (AUC)
Lee et al., 2017	-	1 million	24	Specialty	25.4	[2/3,0,1/3]	×	-	GB	0.832 (AUC)
Alaeddini and Hong, 2017	-	410	-	Specialty	-	5 Fold CV	×	embedded	L1/L2-LR	80 (ACC)
Goffman et al., 2017	-	21,551,572	48	Specialty	13.87	[5/8,0,3/8]	×	wrapper	LR	0.713 (AUC)
Devasahay et al., 2017	410,069	-	11	Specialty	18.59	[1,0,0]	×	embedded	LR, DT	4–23 (Sens)
Harvey et al., 2017	-	54,652	3	Specialty	6.5	[1,0,0]	×	wrapper	LR	0.753 (AUC)
Mieloszyk et al., 2017	-	554,611	192	Specialty	-	5 Fold CV	×	-	LR	0.77 (AUC)
Mohammadi et al., 2018	73,811	73,811	27	Specialty	16.7	10 * [7/10,0,3/10]	×	-	LR, NN, BN	0.86 (AUC)
Srinivas and Ravindran, 2018	-	76,285	-	Primary Care	-	[2/3,0,1/3]	×	-	Stacking	0.846 (AUC)
Ding et al., 2018	-	2,232,737	36	Specialty	13-32	[2/3,0,1/3]	×	embedded	L1-LR	0.83 (AUC)
Elvira et al., 2018	323,664	2,234,119	20	Specialty	10.6	[3/5,1/5,1/5]	×	embedded	GB	0.74 (AUC)
Topuz et al., 2018	16,345	105,343	78	Specialty	-	10 Fold CV	×	embedded	L1-L2-BN	0.691 (AUC)
Chua and Chow, 2019	-	75,677	24	Specialty	28.6	[0.35,0.15,0.50]	×	filter	LR	0.72 (AUC)
Alloghani et al., 2018	-	-	12	-	18.1	[3/4,0,1/4]	×	-	DT, LR	3–25 (Sens)
AlMuhaideb et al., 2019	-	1,087,979	12	Specialty	11.3	10 Fold CV	×	embedded	DT	76.5 (ACC)
Dantas et al., 2019	2660	13,230	17	Specialty	21.9	[3/4,0,1/4]	×	filter	LR	71 (ACC)
Dashtban and Li, 2019	150,000	1,600,000	72	Primary Care	-	[0.63, 0.12, 25]	×	-	NN	0.71 (AUC)
Ahmadi et al., 2019	-	194,458	36	Specialty	23	[70(CV),30]	×	wrapper	GA-RF	0.697 (AUC)
Lin et al., 2019	-	2,000,000	36	Specialty	18	[0.8,0,0.2]	×	embedded	Bay. Lasso	0.70–0.92 (AUC)
Lenzi et al., 2019	5637	40,740	36	Primary Care	13	[1/2,0,1/2]	×	wrapper	MELR	0.81 (AUC)
Praveena et al., 2019	-	100,000	-	Specialty	20	[1,0,0]	×	-	LR, DT	89.6 (ACC)
Li et al., 2019	42,903	115,751	12	Specialty	18	[0.80,0,0.20]	×	-	MELR	0.886 (AUC)
Ahmad et al., 2019	-	10,329	48	Primary Care	-	[2/3,0,1/3]	×	-	Probit R.	0.70 (AUC)
Gromisch et al., 2020	3742	-	24	Specialty	-	[1,0,0]	×	x	LR	75 (Sens)
Aladeemy et al., 2020	-	6599	10	Primary Care	18.58	[0.70,0,0.30]	×	wrapper	SACI-DT	0.72 (AUC)

**Table 3:** Feature selection.

Articles	Patient Demographic													Medical History						Appointment Detail													Patient Behaviour				
	Age	Gender	Language	Race/Etnicity	Employment	Marital Status	Economic Status	Education Level	Insurance/Paym.	ZIP Code	Distance/Transp.	Religion	Access to Phone	Clinic	Specialty	Previous Visits	Provider	Referral Source	Diagnosis	Case Duration	First/Follow-Up	Month	Weekday	Visit Time	Holiday Indicator	Same Day Visit	Weather	Season	Visit Interval	Lead Time	Waiting Time	Scheduling Mode	Prev. No-Show	Prev. Cancel	Last Visit Status	Visit Late	Satisfaction
Dervin et al., 1978	∘	∘				∘			∘				∘						∘		∘									∘		∘					
Dove and Schneider, 1981	∘			×							∘										×									∘		×			∘		
Goldman et al., 1982	∘	×		∘	×				×		×				∘				∘	×										×			∘			×	×
Snowden et al., 1995	×	×	∘	∘		∘	∘			∘	×							×	×	×	∘						×			×		×	∘		×		×
Bean and Talaga, 1995	∘	∘													∘															∘							
Lee et al., 2005	∘	×		∘							×		∘		∘															∘			∘				
Qu et al., 2006	∘								∘												∘						∘			×			∘				
Chariatte et al., 2008	∘	∘																	∘										∘				∘	∘	∘		
Glowacka et al., 2009	∘	∘	×	×	×	∘		∘	∘	∘							∘		×				∘	∘					∘						∘		
Daggy et al., 2010	∘					∘			∘		∘					∘			∘				×	×					∘	∘			∘				
Alaeddini et al., 2011	∘	∘				∘			∘	∘				∘											∘				∘								
Cronin et al., 2013	∘	×	∘						∘												×		∘	×						∘			∘				
Levy, 2013	∘	∘				∘													∘			∘	∘	∘													
Norris et al., 2014	∘								∘														∘	∘			×			∘			∘	∘			
Dravenstott et al., 2014	∘	×			∘	∘			∘	×	∘				×								∘	×				∘	×	∘			∘	∘			
Lotfi and Torres	×	×		×	×			×			×																			×			∘	∘	×		
Huang and Hanauer, 2014	∘	×	∘	∘					∘		∘	×									∘	∘	∘	∘						∘			∘				
Ma et al., 2014	∘	×	∘	∘					×	∘	×		∘		×	∘		∘					∘	×						∘			×				
Alaeddini et al., 2015	∘	∘				∘			∘	∘				∘											∘				∘								
Blumenthal et al., 2015	×	∘						∘	×										∘											∘			∘				
Torres et al., 2015	∘	∘	∘	×		×			∘	∘					∘								∘	∘				∘		∘			∘				
Woodward et al., 2015	×	×		∘															∘														∘				
Peng et al., 2016	∘	∘				∘			∘		×										∘			∘			∘			∘							
Kurasawa et al., 2016	∘	∘									∘								∘				∘				∘			∘							
Harris et al., 2016																																	∘				
Huang and Hanauer, 2016	∘	∘	∘	∘					∘		∘	×									∘	∘	∘	×						×			∘				
Lee et al., 2017	∘	×	×	∘		×	∘		×	×	×	×	×	∘	∘	×	∘	∘	×	∘	×	×	×	×	×	×			∘	∘	×		×	×	∘	∘	
Alaeddini and Hong, 2017	∘	∘				∘	∘		∘		∘												∘	∘													
Goffman et al., 2017	∘	∘				∘															×	∘	∘			∘			×	∘			∘	∘			
Devasahay et al., 2017	∘	∘					∘		∘		∘		∘					∘				∘	∘	∘													
Harvey et al., 2017	∘	∘	×	∘	×	∘	×	∘	∘		×					∘							∘	×						∘			∘				
Mieloszyk et al., 2017	∘	∘					∘								∘							∘	∘	∘						∘		∘					
Mohammadi et al., 2018	∘	×		×	∘	×	×		∘		×		∘		×						×		∘	×				×	∘	∘		×	∘				
Srinivas and Ravindran, 2018	∘	∘		∘		∘			∘	∘	∘										∘			∘			∘			∘	∘						
Chua and Chow, 2019	∘	×		∘										×	∘			∘			∘	∘	∘		∘					∘					∘		
Ding et al., 2018	∘	∘		∘	∘	∘	∘						∘	∘	∘	∘		∘	∘				∘	∘						∘		∘	∘				
Elvira et al., 2018	∘	×												×	∘	∘				∘	×	∘	∘	∘					∘	∘					∘		
Topuz et al., 2018	∘	∘					∘				∘					∘						∘	∘	∘	∘					∘							
Alloghani et al., 2018	∘	∘		∘						∘				∘	∘						∘	∘	×										∘				
AlMuhaideb et al., 2019	∘	∘					∘				∘				∘						∘		∘	∘									∘				
Dantas et al., 2019	×	×							×		∘				∘	∘					∘	∘	∘	∘						∘			∘				
Dashtban and Li, 2019	∘	∘																	∘								∘			∘			∘				
Ahmadi et al., 2019	∘	∘			∘	∘			∘	∘	∘		∘		∘	∘	∘			∘		∘	∘	∘				∘	∘	∘			∘	∘			
Lin et al., 2019	∘	∘		∘			∘		∘				∘																	∘			∘				
Lenzi et al., 2019	∘	∘		∘													∘					∘	∘	∘		∘				∘	∘		∘				
Praveena et al., 2019	∘	∘						∘			∘								∘				∘														
Li et al., 2019	∘			∘		∘			∘								∘						∘				∘						∘	∘			
Ahmad et al., 2019	∘	×		×					∘												∘	×	×										∘				
Gromisch et al., 2020	∘	×				∘					∘		∘			∘			∘	∘	∘																
Aladeemy et al., 2020	×	×				×	×				×		∘								×		∘	×			×		∘	∘			×				

**Table 4:** Performance measures.

Articles	No-Show Rate	AUC	Accuracy	Sensitivity	Specificity	PPV	NPV	Precision	Recall	RMSE	MSE	MAE	F-Measure	G-Measure	TOTAL
Dervin et al., 1978	×		×												1
Dove and Schneider, 1981	×		×									×			2
Goldman et al., 1982	×														0
Snowden et al., 1995	×		×	×	×										3
Bean and Talaga, 1995	×														0
Lee et al., 2005	×	×	×	×	×										4
Qu et al., 2006										×					1
Chariatte et al., 2008															0
Glowacka et al., 2009															0
Daggy et al., 2010	×	×													1
Alaeddini et al., 2011			×								×				2
Cronin et al., 2013	×														0
Levy, 2013	×			×	×										2
Norris et al., 2014	×		×	×	×										3
Dravenstott et al., 2014	×	×	×	×	×	×	×								6
Lotfi and Torres, 2014	×		×	×	×	×	×								5
Huang and Hanauer, 2014	×		×												1
Ma et al., 2014	×		×												1
Alaeddini et al., 2015	×	×									×				2
Blumenthal et al., 2015	×	×	×	×	×	×	×								6
Torres et al., 2015	×	×													1
Woodward et al., 2015	×														0
Peng et al., 2016		×													1
Kurasawa et al., 2016	×	×						×	×				×		4
Harris et al., 2016	×	×													1
Huang and Hanauer, 2016	×	×													1
Lee et al., 2017	×	×						×	×						3
Alaeddini and Hong, 2017		×	×												2
Goffman et al., 2017	×	×													1
Devasahay et al., 2017	×			×	×	×	×								4
Harvey et al., 2017	×	×													1
Mieloszyk et al., 2017		×													1
Mohammadi et al., 2018	×	×	×	×	×										4
Srinivas and Ravindran, 2018		×	×	×	×										4
Ding et al., 2018	×	×													1
Elvira et al., 2018	×	×	×	×	×	×	×								6
Topuz et al.		×	×											×	3
Chua and Chow, 2019	×	×													1
Alloghani et al., 2018	×			×	×	×	×								4
AlMuhaideb et al., 2019	×	×	×	×	×										4
Dantas et al., 2019	×		×	×	×										3
Dashtban and Li, 2019		×	×					×	×						4
Ahmadi et al., 2019	×	×		×	×										3
Lin et al., 2019	×	×													1
Lenzi et al., 2019	×	×													1
Praveena et al., 2019	×		×	×	×			×	×						5
Li et al., 2019	×	×													1
Ahmad et al., 2019		×													1
Gromisch et al., 2020			×	×	×										3
Aladeemy et al., 2020	×	×		×	×										3
TOTAL	38	29	21	17	17	6	6	4	4	1	2	1	1	1	

## Abbreviations

The following abbreviations are used in this manuscript:

ACC	Accuracy
ARM	Association Rule Mining
AUC	Area under the receiver operating characteristic curve
BN	Bayesian Network
BU	Bayesian Update
CHAID	Chi-squared automatic interaction detector
CV	Cross Validation
DT	Decision Tree
EHR	Electronic Health Records
GA	Genetic Algorithm
GB	Gradient Boosting
LD	Linear Discriminant
LR	Logistic Regression
MAS	Mean Absolute Error
MELR	Mixed effects Logistic Regression
MM	Markov model
MSE	Mean Squared Error
NN	Neural Network
NPV	Negative Predictive Value
PRISMA	Preferred Reporting Items for Systematic Reviews and Meta-analyses
PPV	Positive Predictive Value
RF	Random Forest
RMSE	Root Mean Squared Error
SACI	Opposition-based Self-Adaptive Cohort Intelligence
Sens	Sensitivity
SUMER	Sums of exponential for regression
WOS	Web of Science
